# Sexual functioning and marital satisfaction among endometriosis patients in Malaysia: a cross-sectional study

**DOI:** 10.3389/fpsyg.2023.1224995

**Published:** 2023-07-21

**Authors:** Sharvina Ramesh Rao, Luke Woon Sy-Cherng, Abdul Muzhill Hannaan Abdul Hafizz, Mohd Nazzary Mamat @ Yusof, Mohamad Nasir Shafiee

**Affiliations:** ^1^Department of Obstetrics and Gynaecology, Faculty of Medicine, UKM Medical Centre, Kuala Lumpur, Malaysia; ^2^Department of Psychiatry, Faculty of Medicine, UKM Medical Centre, Kuala Lumpur, Malaysia

**Keywords:** sexual functioning, marital satisfaction, endometriosis, MVFSFI, MVGRIMS

## Abstract

Endometriosis affects the sexual functioning and marital satisfaction of couples in a complex manner due to its clinical presentation of the disease. This study aimed to evaluate the prevalence of sexual dysfunction and marital disharmony among women with endometriosis beyond their diagnosis and treatment. A cross-sectional online survey was conducted among women with endometriosis in an endometriosis society at a Malaysian university hospital. Sociodemographic and clinical data were collected. Sexual function was measured using the Malay Version Female Sexual Function Index (MVFSFI), while marital satisfaction was evaluated with the Malay Version Golombok Rust Inventory for Marital Satisfaction (MVGRIMS). A total of 166 patients participated in this survey. The median age was 35 years (Interquartile range, IQR:32.00–39.25 years); 91.6% of participants were Malay. The median score of MVFSFI was 56.00 (IQR: 34.75–68.00). Most of the study subjects (*n* = 96) reported poor to very severe marital satisfaction problems, equivalent to MVGRIMS transformed score of more than 5. High levels of MVGRIMS scores have a moderately strong negative correlation with lower scores for most domains of the MVFSFI. In the stepwise multiple logistic regression, only MVFSFI total scores (*p* = 0.029), MVFSFI lubrication scores (*p* = 0.009), and MVFSFI satisfaction (*p* = 0.010) scores were significantly associated with poor marital satisfaction. Both sexual dysfunction and marital satisfactions commonly affect women with endometriosis and are closely interlinked. Targeted efforts should be made in multiple aspects to improve the quality of sexual functioning and marital satisfaction among endometriosis patients.

## 1. Introduction

Endometriosis is the presence of the endometrium outside the uterine cavity accompanied by chronic inflammation that may cause pain and infertility. This disease was first described by Danial Shroen in 1690 under the work of “Disputatio Inaugralis Medica de Ulceribus Ulceri” (Smolarz et al., 2021). Globally, endometriosis affects roughly 10–15% (190 million) of reproductive-age women and girls (World Health Organization, 2021). Endometriosis can be caused by early menarche, a shorter menstrual cycle, high body mass index (BMI), daily smoking, and daily consumption of at least 10 g of alcohol per day (Smolarz et al., 2021). Clinical symptoms of endometriosis include dysmenorrhea, chronic pelvic pain, dyspareunia, lumbar pain, dyschezia, dysuria, and infertility. Endometriosis affects the patient entirely and could also affect the sexual health of the patient (Pérez-López et al., 2020).

A cross-sectional study revealed that women with endometriosis are approximately three times as likely as women in the general population to develop sexual dysfunction. The fact that endometriosis negatively impacts female sexual function needs to be acknowledged and should be given due importance. It would be an uphill task to recover these women’s global quality of life without adequately addressing the sexual aspects (Fairbanks et al., 2017). A meta-analysis conducted across PubMed-Medline, Scopus, EMBASE, and Web of Science databases, showed that there were significantly lower scores for each of the domains of the Female Sexual Function Index (FSFI) found in women with endometriosis which strongly points toward the notion of endometriosis impairs their sexuality (Pérez-López et al., 2020). A case–control study in China revealed that endometriosis significantly affected multiple aspects of sexual function, where their FSFI were lower than the control group of patients. The main manifestations of sexual dysfunctions in women with endometriosis were difficulty in subjective arousal, poor vaginal lubrication during sexual activity, sexual pain, and decreased satisfaction with sexual life (Yang et al., 2021).

A search of the literature conducted to determine the quality of life of women with endometriosis revealed that endometriosis has an effect on their quality of life. The majority of individuals reported dysmenorrhea, dyspareunia, and dyschezia as endometriosis-related symptoms, but only 39.6% of people experienced dysuria. The mean health-related quality of life (HRQoL) score was 41.8 with a range of 0–86.7, whereas the mean score of Sexual Quality of Life (SQoL) for the total study population (*n* = 192) was 47.5 with a range of 0–100 (van Poll et al., 2020). Thus, this clearly shows that endometriosis affects the patient’s quality of life, which could also signify poor marital satisfaction.

A systematic review done to review the effects of endometriosis on sexuality and a couple’s relationship exhibited that dyspareunia experienced by women with endometriosis leads to anxiety and depression that impacts the female sexual function and marital relationship. All the studies reviewed by this systematic review came to a similar conclusion that dyspareunia in endometriosis patients impacts their psychological viewpoint of sexuality and intimacy, which in turn affects their relationship with their spouse (Norinho et al., 2020). Sexual functioning and marital satisfaction play an important role in a woman’s life. Endometriosis affects a woman in many ways other than affecting their health. Through this research, we propose to dive deeper into understanding the depth and severity of sexual dysfunction and marital dissatisfaction faced by women having endometriosis. The main objective of this study was to evaluate the prevalence of sexual dysfunction and marital disharmony among endometriosis patients beyond their diagnosis and treatment. The secondary objective was to determine the relationship between sexual function and marital harmony. We hypothesized that greater sexual dysfunction is associated with increased marital disharmony among women with endometriosis.

## 2. Methodology

This study was a cross-sectional online survey in a study population of women diagnosed with endometriosis. It was conducted at Universiti Kebangsaan Malaysia Medical Centre (UKMMC) from January 2022 until June 2022. Participants were recruited using convenience sampling and needed to respond to the study questionnaires via a Google Forms link sent to the participating patients.

The sample size for this study was based on a study done by Mishra et al. that evaluated the association between endometriosis and female sexual dysfunction and marital relationship using the Female Sexual Function Index (FSFI) questionnaire (Mishra et al., 2016). This study included 51 participants clinically diagnosed with endometriosis, with the prevalence of female sexual dysfunction affecting marital relationships, was 47.06% (*n* = 24). The sample size for the research was calculated using the formula for prevalence study (with finite population correction) with the precision (d) = 0.05. The sample size (*n*) obtained was 382. The inclusion criteria for this study were: Malaysian, legally married, aged 18 years or older, completed the primary treatment of endometriosis for at least 3 months after the initial diagnosis was told to the patient, physically independent, and able to read the questionnaire. The exclusion criteria, on the other hand, were significant amnesia, physically dependent, and usage of drugs that may suppress sexual desire, such as antidepressants and anxiolytics.

Women with endometriosis were approached via the endometriosis support group at UKMMC and were recruited in this study following written consent through the Google form link. The participants will be kept anonymous, and no details will be disclosed. Two structured questionnaires were given using a Google form link: the Malay Version of the Female Sexual Function Index (MVFSFI) and the Malay Version Golombok Rust Inventory for Marital Satisfaction (MVGRIMS).

The MVFSFI is a brief, valid, and reliable self-report measure of female sexual function developed and cross-validated by Rosen et al. in 2000 (Rosen et al., 2000). This questionnaire covers six basic domains of female sexual dysfunction: desire, subjective arousal, lubrication, orgasm, satisfaction, and pain. This questionnaire was validated in a cross-sectional study conducted on the Malaysian population (Sidi et al., 2007). This index was a good tool for measuring sexual dysfunction among Malaysian women. It does not contain too intimate or embarrassing questions on sexual matters. The translated questionnaire’s reliability analysis revealed a high test–retest correlation among respondents (Pearson correlation for at least r < 0.7). Based on another reliability study, the item reliability index indicates Cronbach’s Alpha’s internal consistency as it revealed each domain’s scale value in the study as more than 0.6 (Jackson and Furnham, 2000). A total score ≤ 55 and above was found to be the appropriate cut-off point to distinguish between women with sexual dysfunction and those without (sensitivity of 99% and specificity of 97%) compared to the original version of FSFI. The lower the scores, the higher likelihood the women would suffer from sexual dysfunction. The cut-off scores for each domain were also established, where each domain had different cut-off scores. For example, sexual desire disorder had a cut-off score of ≤5, sexual arousal disorder had a cut-off score of ≤9, and disorder of lubrication had a cut-off score of ≤10.

The second part of the questionnaire was based on MVGRIMS. The Golombok-Rust Inventory of Marital State (GRIMS) has become the most used instrument in multicenter and international clinical trials to assess marital satisfaction since its first introduced in 1986 (Rust et al., 1986). The GRIMS is easy and short to be administered to evaluate the state of marriage and relationship (Rust et al., 2010). It contains 28 4-point Likert scale items (‘Strongly disagree’ score 0, ‘Disagree’ score 1, ‘Agree’ score 2, and ‘Strongly agree’ score 3). A raw score is calculated according to the scoring guide and then transformed into a standardized GRIMS score. A higher score represents a more problematic relationship, with a transformed score of 5 representing an average relationship (Rust et al., 2010). A score of 9 indicates very severe problems, whereas a score of 2 represents a very good relationship. The authors caution against interpreting a score of 1 as responses resulting in this score might be unreliable (Rust et al., 2010) and validated the MVGRIMS in a Malaysian population in 2002 (Quek et al., 2002). The MVGRIMS displayed excellent internal consistency (Cronbach’s alpha value = 0.59 to 0.91), and the test–retest and intra-class correlation coefficients were highly significant in most items (ICC = 0.62 and above). There was also high sensitivity and specificity (Quek et al., 2002).

Statistical analysis was conducted using the Statistical Package for Social Science version 26.0 (IBM Corp., Armonk, NY, USA). Descriptive statistics of the study subjects were generated. The normality test showed that the continuous data were normally distributed (Kolmogorov–Smirnov test, *p* < 0.05). Categorical variables were reported in frequency and percentage, while continuous variables were in the median and interquartile range (IQR). The correlation between the MVGRIMS score and MVFSFI total and sub-scores was measured using Spearman’s correlation coefficient. MVGRIMS scores were divided into two categories. A score of <5 represented better marital satisfaction, and a score of >5 represented worse marital satisfaction. Bivariate analysis was run to examine the differences between these two groups regarding demographic, social, and clinical characteristics, using the chi-square test or Fisher’s exact test for categorical variables and Mann–Whitney U-test for continuous variables. Significant variables were then included in a stepwise multiple logistic regression model as independent variables to look for factors significantly associated with marital dissatisfaction. The logistic regression model displayed a good fit with a non-significant Hosmer-Lemeshow goodness-of-fit test (*p* = 0.632). All statistical tests’ significance level (alpha) was set at *p* < 0.05.

Ethics approval (code: JEP-2021-914) was reviewed and granted by the Research Ethics Committee, The National University of Malaysia for this study before its commencement. The research project was registered under the Secretariat of Research and Innovation, Faculty of Medicine, The National University of Malaysia (Project Code: FF-2022-046). Before commencing the study, the EQUATOR (Enhancing the QUAlity and Transparency Of health Research) Network guidelines were adhered to.

## 3. Results

A total of 166 subjects participated in this study and completed the questionnaires. The sociodemographic and clinical characteristics of the study subjects are shown in Table 1. The median age of the subjects was 35 years old, with an interquartile range of 32.00 to 39.25 years old. Most participants were Malays, consisting of 91.6% of the total participants, followed by 2.4% of Chinese, 3.0% of Indians, and 3.0% of other ethnicities. Among the participants of this study, 95.8% of them were married, 1.8% were unmarried, and the remaining 2.4% were separated, or their partners had passed away. Almost half of the participants (48.2%) were diagnosed with severe endometriosis, whereas 24.1% were unsure of the severity of their diagnosis, 18.1% were diagnosed with moderate severity of endometriosis, and the remainder 9.1% had mild endometriosis. Most of our participants received a combination of medical and surgical treatment without undergoing additional fertility treatments. The median score of the MVFSFI was 56.00, with an interquartile range score of 34.75 to 68.00. The MVFSFI scores were further broken down into domains, which were desire (median = 6, IQR: 4–6), arousal (median = 12, IQR: 7–14), lubrication (median = 13, IQR:8–16), orgasm (median = 9, IQR: 4–12), satisfaction (median = 9, IQR: 5–12) and pain (median = 7, IQR: 5–8).

**Table 1:** Sociodemographic and clinical characteristics of the study subjects (*N* = 166).

Variable	*n* (%)^a^	Median (IQR)^b^
Age (years)		35 (32.00–39.25)
Ethnicity		
Malay	152 (91.6)	
Chinese	4 (2.4)	
Indian	5 (3.0)	
Others	5 (3.0)	
Marital status		
Unmarried	3 (1.8)	
Married	159 (95.8)	
Separated/ partner passed away	4 (2.4)	
No. of children		
Have children	64 (38.6)	
No children	102 (61.4)	
Severity of endometriosis		
Mild	16 (9.6)	
Moderate	30 (18.1)	
Severe	80 (48.2)	
Unsure	40 (24.1)	
Treatment received		
Medical	21 (12.7)	
Surgical	18 (10.8)	
Combination (without fertility treatment)	84 (50.6)	
Combination (with fertility treatment)	36 (21.7)	
No treatment received	7 (4.2)	
MVFSFI total score		56 (34.75–68.00)
Domains of MVFSFI		
Desire		6 (4–6)
Arousal		12 (7–14)
Lubrication		13 (8–16)
Orgasm		9 (4–12)
Satisfaction		9 (5–12)
Pain		7 (5–8)

^a^Number of case (Percentage); ^b^Median (Interquartile range).

The participants’ marital satisfaction levels were classified based on the MVGRIMS transformed scores. Over half of the participants had poor to very problematic marital satisfaction. Most of them involved in this study had poor marital satisfaction (19.3%), followed by bad marital satisfaction (14.5%), but surprisingly several participants reported very good marital satisfaction (11.4%). 10.2% of participants experienced severe problems in their marital life, whereas an equal number of participants had above-average and average marital satisfaction. A small group of participants had severe marital satisfaction problems (7.8%). The levels of marital satisfaction among participants were further grouped into two major groups where the participants with better marital satisfaction consisted of participants with average to very good MVGRIMS transformed scores (*n* = 70). In contrast, worse marital satisfaction consisted of participants with poor to very severe problems (*n* = 96), as seen in Table 2.

**Table 2:** Levels of marital satisfaction among the subjects (*n* = 166).

Variable	*n*	Percentage
Very good	19	11.4
Good	15	9.0
Above average	18	10.8
Average	18	10.8
Poor	32	19.3
Bad	24	14.5
Severe problems	17	10.2
Very severe problems	13	7.8
Score of “1”	10	6.0

The relationship between MVGRIMS transformed scores and MVFSFI total scores ereanalyzed using the Pearson correlation coefficient. Preliminary analyses were performed to ensure no violation of the assumptions of normality and linearity. Both the scores displayed a normal distribution. There was a moderately strong negative correlation between the two variables (*r* = −0.414, *p* < 0.001, with 95% confidence interval (C.I) −0.526 to −0.273) with high levels of MVGRIMS transformed scores associated with lower levels of MVFSFI total scores. The Pearson Correlation coefficient demonstrated that the domains of desire, arousal, lubrication, orgasm, and satisfaction in MVFSFI were significantly correlated with MVGRIMS transformed scores. Only one domain in the MVFSFI, the pain domain, exhibited no significant correlation with the MVGRIMS transformed scores (*r* = −0.017, *p* = 0.827 with 95% C.I. −0.177 to 0.147) (Table 3).

**Table 3:** Correlations between MVGRIMS transformed score and MVFSFI scores (*n* = 166).

Variable	Spearman’s rho	Value of *p*
MVFSFI desire	−0.311	<0.001*
MVFSFI arousal	−0.408	<0.001*
MVFSFI lubrication	−0.404	<0.001*
MVFSFI orgasm	−0.367	<0.001*
MVFSFI satisfaction	−0.415	<0.001*
MVFSFI pain	−0.146	0.06
MVFSFI total score	−0.428	<0.001*

^*^Statistically significant.

Comparisons between study participants with better and worse marital satisfaction were done using the Chi-square test for categorical variables, whereas the Mann–Whitney U test was for continuous variables. The chi-square test indicated no significant association between the variables: ethnicity, marital status, number of children, the severity of endometriosis, and treatment received with marital satisfaction. The Mann–Whitney U test revealed no significant comparison between age and marital satisfaction, whereas it exhibited a significant difference in the MVFSFI total scores and marital satisfaction, as shown in Table 4.

**Table 4:** Comparisons between study subjects with better and worse marital satisfaction (*n* = 156).

Variable	Marital satisfaction				Value of *p*
	Better		Worse		
	*n* (%)	Median	*n* (%)	Median	
Age (years)	35.00		36.00	0.146 ^a^	
Ethnicity					
Malay	63 (90.0)		79 (91.9)		0.284^b^
Chinese	2 (2.9)		2 (2.3)		
Indian	1 (1.4)		4 (4.7)		
Others	4 (5.7)		1 (1.2)		
Marital status					
Unmarried	2 (2.9)		1 (1.7)		0.145^b^
Married	68 (97.1)		81 (82.1)		
Separated/ partner passed away	0 (0.0)		4 (2.2)		
No. of children					
Have children	22 (31.4)		48 (68.6)		0.137^b^
No children	37 (43.0)		49 (57.0)		
Severity of endometriosis					
Mild	10 (14.3)		4 (4.7)		0.159^b^
Moderate	10 (14.3)		18 (20.9)		
Severe	34 (48.6)		41 (47.7)		
Unsure	16 (22.9)		23 (26.7)		
Treatment received					
Medical	8 (11.4)		13 (15.1)		0.195^b^
Surgical	5 (7.1)		11 (12.8)		
Combination (without fertility treatment)	32 (45.7)		45 (52.3)		
Combination (with fertility treatment)	20 (28.6)		15 (17.4)		
No treatment	5 (7.1)		2 (2.3)		
MVFSFI scores^a^					
Desire		6.0		5.0	0.041*^a^
Arousal		12.0		10.0	0.002*^a^
Lubrication		15.0		12.0	<0.001*^a^
Orgasm		10.0		8.0	0.002*^a^
Satisfaction		10.5		7.5	0.001*^a^
Pain		7.0		7.0	0.446^a^
Total		62.0		50.5	<0.001*^a^

^a^Mann–Whitney U test; ^b^Chi-square test. *Statistically significant.

Thus, only MVFSFI total scores, desire, arousal, lubrication, orgasm, and satisfaction were included in the stepwise multiple logistic regression analysis. It was found that the MVFSFI total scores, MVFSFI lubrication scores, and MVFSFI satisfaction scores were found to be significantly associated with poor marital satisfaction, as shown in Table 5. Thus, sexual dysfunction does affect the marital satisfaction of women with endometriosis.

**Table 5:** Logistic regression analysis for factors associated with worse marital satisfaction.

	B	S.E.	Wald	df	*p*	Odds ratio	95.0% C.I. for Odds Ratio	
							Lower	Upper
MVFSFI total scores	−0.217	0.100	4.772	1	0.029*	0.805	0.662	0.978
MVFSFI desire	0.165	0.157	1.114	1	0.291	1.180	0.868	1.604
MVFSFI arousal	0.174	0.143	1.472	1	0.225	1.190	0.899	1.575
MVFSFI lubrication	0.365	0.140	6.844	1	0.009*	1.440	1.096	1.893
MVFSFI orgasm	0.153	0.160	0.908	1	0.341	1.165	0.851	1.595
MVFSFI satisfaction	0.387	0.149	6.713	1	0.010*	1.472	1.099	1.973

*x*^2^ = 2.214, df = 1, *p* < 0.137; Nagelkerke *R*^2^ = 0.019. ^*^Statistically significant.

## 4. Discussion

The present study aimed to evaluate the prevalence of sexual dysfunction and marital disharmony among endometriosis patients beyond their diagnosis and treatment. It was done by collecting responses from 166 participants via two structured questionnaires, the MVFSFI, and the MVGRIMS. This study discovered that lower MVFSFI scores (indicating a greater degree of sexual dysfunction) are significantly associated with poor marital satisfaction, as defined by the MVGRIMS transformed score of more than 5. Further analysis is done through the stepwise multiple logistic regression analysis further showed a significant association between sexual dysfunction and marital dissatisfaction.

Based on the Diagnostic and Statistical Manual of Mental Disorders (DSM American Psychiatric Association [APA]), sexual dysfunction is defined as “a clinically significant disturbance in a person’s ability to respond sexually or to experience sexual pleasure” (Mitchell et al., 2016). A case–control study done in China by Yang et al. demonstrated significant differences in the quality of sexual life between women with endometriosis and healthy women (Yang et al., 2021). Their findings showed that women with endometriosis had trouble with subjective arousal, had poor vaginal lubrication during sexual activity, had sexual pain, and decreased satisfaction with sexual life. As our study also used a similar mode of evaluation of sexual function through the MVFSFI, it reflects similar findings as the study done in China. Women with endometriosis in Malaysia, as represented through this experience reduced sexual function in all domains of female sexual function in which they have reduced desire, reduced arousal toward sexual activity, poor vaginal lubrication, absence of orgasms, sexual dissatisfaction, and presence of dyspareunia.

Marital satisfaction is an indicator of the marriage’s quality, where the couple’s genuine pleasure, satisfaction, and joyfulness are experienced when they consider all aspects of marriage (Taghani et al., 2019). In short, marital satisfaction reflects the married couple’s feeling of contentment towards one another in all aspects of marriage. According to a study conducted in Portugal, greater marital happiness is strongly associated with greater sexual pleasure among women with endometriosis; these two factors are considerably correlated (Martins et al., 2022). Martins et al. (2022) exhibited that having received surgical treatments alone may not be likely to solve the issues related to marital satisfaction among endometriosis patients. Surgical procedures have become more challenging in conditions such as deeply infiltrating endometriosis; therefore, the application of surgical neuroanatomic principles (i.e.: neuropelveology) for the diagnosis and treatment should be considered to ensure optimal radicality and minimally invasive surgery that will advantageous to the patient’s condition in terms of reproducible, safety and effectiveness (Raffaelli et al., 2018; Di Donna et al., 2021a,b). As a person with endometriosis may develop menopause symptoms such as vaginal atrophy, the fractional micro-ablative CO2 laser is a therapeutic option that can improve vaginal health and endometriosis-related symptoms (D'Oria et al., 2021; Woźniak et al., 2023). Hence, a comprehensive endometriosis team in surgical management is required and researched to ensure long-term results in pelvic discomfort, recurrence rate, and fertility, while dramatically enhancing endometriosis patient’s quality of life and sexual performance (D'Alterio et al., 2021).

Another study revealed that increased sexual activity and marital happiness contributed to greater sexual satisfaction (Pereira et al., 2021). This study indicated that women with endometriosis have poorer marital satisfaction as a result of reduced sexual activity and impaired sexual satisfaction, which is mostly caused by discomfort during intercourse. Contrary to this study, our study exhibited that dyspareunia was not significantly associated with marital satisfaction. It could be postulated that although dyspareunia plays a very important role in the quality of life of women with endometriosis, the cultural limitations could hold back a woman’s expression of sexual pain. A multi-country case–control study revealed that French women without endometriosis were more likely to experience deep dyspareunia than Chinese women with endometriosis (Chapron et al., 2016). This was likely related to differences in how women from various ethnic and societal backgrounds conceptualize pain. Hence, Malaysian women rich in their cultural practices might conceptualize dyspareunia from a different perspective, leading to dyspareunia not contributing to marital satisfaction.

This study presents certain limitations, such as this mode of cross-sectional study design does not allow temporal relationships to be established; uses self-report questionnaires. Future research should be designed as longitudinal studies to evaluate women with endometriosis at various stages. They are evaluated depending on the treatment they have already received, which may affect marriage satisfaction and sexual dysfunction outcomes. Another limitation of this study is where it did not include important factors such as personality traits. A systematic review and meta-analysis have shown a negative association between marital happiness and neuroticism (Sayehmiri et al., 2020). Thus, marital satisfaction does not solely depend on the sexual satisfaction of the participant, but the personality of the participant must be considered too. This study also had a few strengths as it is one of the pioneer studies done to analyze sexual dysfunction among endometriosis patients in Malaysia. A study was conducted in Malaysia to examine sexual dysfunction in gynecological cancer survivors (Mohamad Muhit et al., 2022). This study had a similar disadvantage to ours in that the sociocultural context posed a barrier to a comprehensive analysis of sexual dysfunction since Asian women are more reticent to communicate their sexual desires or complaints publicly, especially in front of the public.

## 5. Conclusion

The outcomes of this study on sexual dysfunction and marital satisfaction among endometriosis patients provide more and better information for improving the quality of life of endometriosis patients. According to our research, sexual dysfunction and marital dissatisfaction are significant obstacles in the life of an endometriosis patient. Medical institutions should adopt a new strategy incorporating psychological factors in managing endometriosis patients. As a result, clinicians will be able to identify the impact of sexual dysfunction on the quality of their marital life. Community-based interventions could also play an essential role by establishing support groups that encourage peer-to-peer support and bolstering education about the need for endometriosis patients to share their obstacles.

## Data availability statement

The original contributions presented in the study are included in the article/supplementary material, further inquiries can be directed to the corresponding author.

## Ethics statement

The studies involving human participants were reviewed and approved by Research Ethics Committee, The National University of Malaysia (Reference code: JEP-2021-914). The research project was approved by the Faculty of Medicine, The National University of Malaysia (Project Code: FF-2022-046). The patients/participants provided their written informed consent to participate in this study.

## Author contributions

SR, LS-C, and MS contributed to the conception and design of the study. SR, AH, MM, and MS developed and organized the questionnaire. SR organized the database, performed the statistical analysis, and wrote the first draft of the manuscript. SR, AH, and MS wrote sections of the manuscript. AH and MS reviewed and edited the final manuscript. All authors contributed to the manuscript’s revision and read and approved the submitted version.
